# Therapeutic outcome and long-term naturalistic follow-up of female adolescent outpatients with AN: clinical, personality and psychopathology evolution, process indicators and outcome predictors

**DOI:** 10.1186/s12888-023-04855-0

**Published:** 2023-05-25

**Authors:** Federico Amianto, Luca Arletti, Serena Vesco, Chiara Davico, Benedetto Vitiello

**Affiliations:** 1 Department of Neuroscience, Section of Child and Adolescent Neuropsychiatry, University of Turin, Via Cherasco, 15 - 10126, Turin, Italy; 2 Department of Public Health and Pediatric Sciences, Section of Child and Adolescent Neuropsychiatry, University of Turin, Turin, Italy; 3 Department of Pathology and Care of the Children, Regina Margherita Hospital, Turin, Italy

**Keywords:** Anorexia nervosa, Adolescents, Multimodal treatment, Personality, Psychopathology, Outcome predictors

## Abstract

**Background:**

Anorexia nervosa (AN) is a serious mental illness of growing prevalence in childhood and adolescence. Despite its severity, there are still no completely satisfactory evidence-based treatments. Follow-up studies represent the most effective attempt to enlighten treatment effectiveness, outcome predictors and process indicators.

**Methods:**

Seventy-three female participants affected with AN were assessed at intake (T0) and at 6 (T1) and 12 (T2) months of an outpatient multimodal treatment program. Nineteen participants were assessed 15 years after discharge (T3). Changes in diagnostic criteria were compared with the chi-square test. Clinical, personality and psychopathology evolution were tested with ANOVA for repeated measures, using the t-test or Wilcoxon test as post-hoc. T0 features among dropout, stable and healed participants were compared. Healed and unhealed groups at long-term follow-up were compared using Mann-Whitney U test. Treatment changes were correlated to each other and with intake features using multivariate regression.

**Results:**

The rate of complete remission was 64.4% at T2, and 73.7% at T3. 22% of participants maintained a full diagnosis at T2, and only 15.8% at T3. BMI significantly increased at each time-point. A significant decrease of persistence and increase in self-directedness were evidenced between T0 and T2. Interoceptive awareness, drive to thinness, impulsivity, parent-rated, and adolescent-rated general psychopathology significantly decreased after treatment. Lower reward dependence and lower cooperativeness characterized the dropout group. The healed group displayed lower adolescent-rated aggressive and externalizing symptoms, and lower parent-rated delinquent behaviors. BMI, personality and psychopathology changes were related with each other and with BMI, personality and psychopathology at intake.

**Conclusion:**

A 12-months outpatient multimodal treatment encompassing psychiatric, nutritional and psychological approaches is an effective approach for the treatment of mild to moderate AN in adolescence. Treatment was associated not only with increased BMI but also with positive personality development, and changes in both eating and general psychopathology. Lower relational abilities may be an obstacle to healing. Approaches to treatment resistance should be personalized according to these finding.

## Introduction

Anorexia Nervosa (AN) is a serious mental illness with an adolescence onset in 85% of cases [1]. Current trends evidence an increase of inpatient admissions in childhood [1]. Often it produces severe clinical and social impact in adulthood due to its long and severe course, associated with high rates of chronicity, mortality and relapse [2–7]. In fact, even though numerous follow-up studies on AN are hampered by high dropout rates [8–10], low sample width [6], and a lack of consistency about definitions of recovery, remission and relapse [11, 12], treatment outcomes are generally not completely unsatisfactory.

According to the bio-psycho-social pathogenesis of the disorder, the treatment of anorexia nervosa requires a multidisciplinary approach. This generally includes psychiatric visits with possible drug prescription, psychotherapy sessions, and nutritional care (with dietician and dietitian visits), coupled with family counselling or family psychotherapy [6, 13–15]. These are delivered in different therapeutic settings encompassing inpatient, day hospital (DH), or outpatient services, with different rates of remission according to the heterogeneity of the sample selection [16].

Studies of the short-term outcomes after an inpatient treatment have reported rates of complete remission ranging from 24 to 30% [17–21] to almost 50% [22], rising to 90% if also partial remission is included [19]. In general, improvement rates increase with longer follow-up periods, because the disorder requires long-lasting treatments [23, 24]. In a nutshell, the inpatient treatment displays favorable rates of complete remission up to 70% [6].

With DH treatment, rates of remission which are similar to those of inpatient treatment have been reported [25], ranging from a 40–49% rate of complete remission [26, 27] to 71–78% if also partial remission is considered [28–30].

Outpatient treatment is a cost-effective alternative to inpatient and DH treatments, in particular for those patients who do not require intensive nutritional care [16]. In a randomized study, the rate of remission in outpatient setting has been found lower than in DH [31]. Nevertheless, when the results are controlled for the clinical conditions, the rates of remission are similar to those seen in inpatient settings for patients with full diagnosis of AN, and even greater for atypical AN [32]. Also, the rate of remission in outpatient care increases with a longer follow-up, ranging from 19% at one year to 33% at 2 years, until 64% at 5 years [12, 16]. The overall rate of long-term remission is favorable, with a 90% of stable weight restoring in those who completed the treatment program [33]. Thus outpatient treatment can be considered a first-line treatment setting for AN with a recent onset and better clinical and psychopathological conditions [34], with the convenience of a lower dropout rate with respect to inpatient treatment [35].

A direct comparison between the efficacy of the treatment delivered in the different settings of care is not possible because of the differences in sample selection. Nevertheless, the outpatient setting is the less intensive, thus clinicians may believe that it produces less deep and long-lasting effects on personality and psychopathology of AN adolescents.

Most of the follow-up studies of AN in adolescence focus on objective symptoms such as body weight [36, 37], while data on psychopathology and personality traits are often neglected, thus reducing our understanding of the mechanisms and predictors of improvement. In fact, psychopathology and personality traits along with emotional and behavioral aspects are often neglected, despite evidence that they may reduce treatment efficacy and increase relapse risk [15, 38–41]. An overall assessment of the functioning of the AN participants which includes personality and psychopathology measures is relevant for a deeper understanding of the course of their disorder [42] and for a stronger inference on their outcome [13, 36, 43]. In this regard some literature findings may be of interest. For instance, it was evidenced in the adult population that the overall improvement of AN participants at a 8-year follow-up was associated with a significant reduction in their traits of harm avoidance and with an increase in self-directedness [13], while stable improvement without diagnostic crossover were linked to self-directedness development [44]. Moreover, those AN participants with sustained recovery at a 10-year follow-up had lower levels of maturity fears [20], and were free of other psychopathology [45]. Those with persistent comorbidity manifested worse social interactions and poorer outcomes in adulthood [46], while those who worsened displayed higher drive to thinness and body dissatisfaction [13]. Finally, BMI changes which are reached after the treatment were generally maintained at follow-up [47], but it is unknown if the same happens for psychopathology. It emerges that a further exploration of personality and psychopathology changes during treatment and at long-term follow-up are needed for understanding AN in adolescence.

Our study provides a prospective double assessment (at 6 and 12 months) of personality and psychopathology treatment outcomes and a 15-years naturalistic follow-up of a sample of adolescents with AN treated with an integrated multidisciplinary treatment model. The outcome measures of the present research encompass BMI, eating attitudes and diagnostic criteria, personality traits, eating psychopathology, along with general functioning and general psychopathology assessed by both patients and their parents. The statistical analysis explores the changes between the time-points and the differences among outcome subgroups. The hypothesis is that all outcome measures improve after treatment and some of them also after the long-term follow-up period. Moreover, personality, eating, and general psychopathology differences at T0 may predict treatment outcome and long-term clinical conditions.

## Methods

The present study includes two parts: the first is an assessment of the treatment outcomes in the short (6-months) and medium (12-months) term, and the second is a 15-year long-term follow-up of the treated sample.

It was considered for recruitment in the study a sample of 149 female adolescent outpatients from the Child Neuropsychiatry Outpatient Service for the Eating Disorders the Regina Margherita Hospital, Department of Public Health and Pediatric Sciences of the University of Turin, first evaluated between January 1st, 2003 and December 31st, 2005 (T0 time of this study) who received a full diagnosis of AN (Restricter – ANR or Bingeing-purging –ANBP) or Eating Disorder Not Otherwise Specified (EDNOS AN type) according to diagnostic criteria of DSM IV and IV-TR. The diagnosis was established by a child neuropsychiatrist during the first examination at the intake in the center (T0) using the Structured Clinical Interview for Diagnostic and Statistical Manual of Mental Disorders, Revised Third Edition (SCID-I) [48]. It was revised by another expert child neuropsychiatrist at the follow-up 15 years later (T3) using the SCID-5-CV for DSM 5 criteria [49], thus the study was conducted according to the DSM 5 criteria.

The inclusion criteria were: (1) female adolescents with a diagnosis if AN enrolled for a 1-year treatment at the outpatient clinic for eating disorders (males were not included because they were infrequently seen at the clinic); (2) patients filled in the psychopathology and personality measures at intake in the therapeutic program; (3) absence of documented intellectual disability or neurodevelopmental disorder at intake, defined according to the DSM criteria; (4) absence of psychosis or bipolar disorder at any time-point of the treatment .

**Fig. 1:**
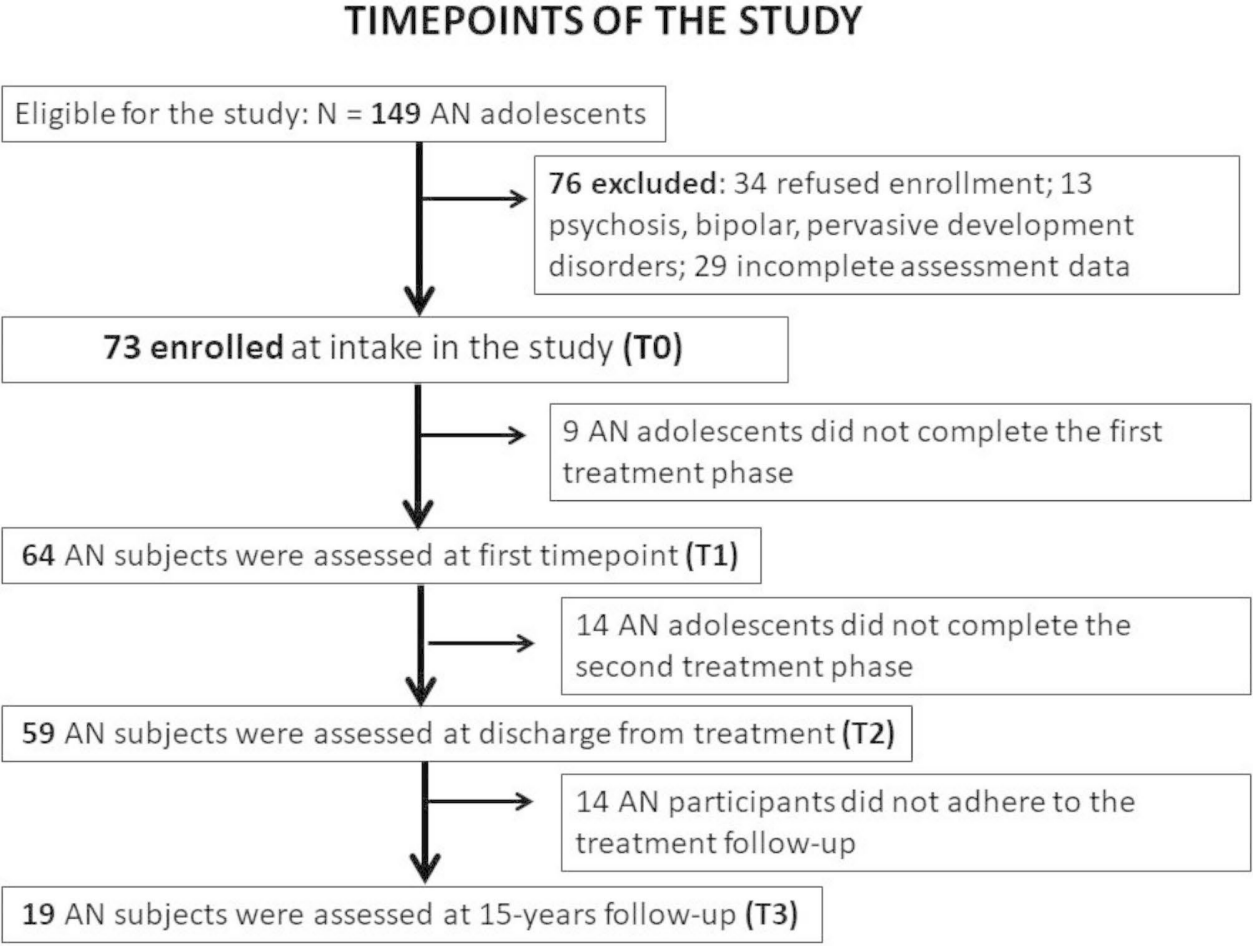
Flow-chart at each treatment time-point

Among 149 eligible subjects, 34 refused enrollment in the study, 13 received a diagnosis of psychosis, pervasive development disorder or bipolar disorder during assessment phase, and in 29 cases the results of the assessment collected for the study were lacking or incomplete. Finally, 73 female adolescent participants were enrolled in the study (mean age: 15.57 *±* 1.33 y; mean age of onset: 15.00 *±* 1.58 y; mean education: 9.25 *±* 1.84 y). Figure 1 displays the flow-chart at each treatment time-point.

At the long-term follow-up, 15 years after treatment discharge, 31 subjects could not be contacted (for 21, the parents refused to give their telephone number to the researchers for privacy protection). Among the remaining 43 subjects, 16 refused to take part in the follow-up assessment for personal reasons, and 8 agreed to participate, but did not return the assessment package with the tests or filled them incorrectly. In conclusion, 19 subjects with AN participated in the 15-year follow-up study. DSM 5 criteria were applied to assess diagnostic outcome at follow-up, using the SCID-5-CV administered by an expert child neuropsychiatrist (FA author).

### Ethical issues

All participants and their parents gave written informed consent for participation in clinical research at intake in the outpatient service. All contacted participants who agreed to participation in the follow-up study gave written informed. The Intercompany Review Board of Torino (CEI) approved this study with the protocol number 36,931.

### Treatment approach

All selected participants were voluntarily enrolled for a 1-year treatment at the outpatient service for Eating Disorders of the Regina Margherita Hospital in Turin. The treatment program was multidisciplinary and included: a first assessment visit conducted by an experienced child psychiatrist assisted by a specializing doctor, self-administration of assessment instruments at home, a second assessment visit in which the results of the tests, modality and timing of the program were explained to the participant and to her parents and possible drug prescription (antidepressant or anxiolytic). Neuropsychiatric control visits were conducted once a month. First joint visit by a dietician and a dietitian with a diet-therapy prescription followed by diet-therapy follow up once/month. Assignment to two cycles of time-limited (20 sessions), weekly brief psychodynamic psychotherapy (specifically Adlerian Psychodynamic Psychotherapy, B-APP). Therapists received a supervision according to this psychotherapeutic approach according to the manualized model of Fassino and coworkers [50].

The participants included in the present study did not receive any psychiatric prescription drug, but only nutritional supplements as when necessary.

### Outcome measures

ED diagnosis according to DSM 5 (AN Restricting type - ANR, AN Bingeing-purging type - ANBP, Other Specified Feeding or Eating Disorders - OSFED) and clinical data (height, weight, BMI, bingeing/purging per week, hours of physical activity per week, and minor psychiatric symptoms) of participants were collected at their first access into the center (T0) and at the time points of clinical outcome and follow-up. In consideration of their relevance for the study, of the diagnostic composition of the final sample, and data missing they were considered as clinical outcome measures ED diagnosis and BMI.

The time points were defined at intake (T0), at 6 months after the beginning of the treatment (T1), at 12 months corresponding to the end of the treatment program (T2), and at 15-years follow-up (T3).

At T0 and T2 participants were assessed with a battery of psychometric tests including: Temperament and Character Inventory (TCI) [51], Eating Disorder Inventory-2 (EDI-2) [52], Young Self-report Questionnaire (YSR); while their parents filled in the Child Behavior Checklist (CBCL).

At T1 only the Eating Disorder Inventory-2 (EDI-2), Young Self-report Questionnaire (YSR), and the Child Behavior Checklist (CBCL) were administered because the changes in personality traits within six months were considered unstable [51]. The TCI was not administered at this time-point because the possible changes in personality traits were considered unstable due to the short timeframe.

At T3 the Temperament and Character Inventory (TCI) and the Eating Disorder Inventory-2 (EDI-2) were administered because the CBCL and the YSR are measures validated only for minors.

#### Materials

All the participants were administered the same battery of psychometric tests including:

The *Temperament and Character Inventory (TCI)*, which provides a clinical classification of different personality traits according to the Cloninger model [53]. It includes four temperament subscales: Novelty Seeking, Harm Avoidance, Reward Dependence, Persistence, and three character subscales: Self-directedness, Cooperativeness, Self-transcendence. Each dimension is represented by sub-dimensions which better specify the meaning of the scale, nevertheless for the aims of the present research only main dimensions were used. Concerning reliability and validity, its psychometric properties support its clinical usefulness in the assessment of personality psychopathology [54]. Cronbach’s alpha for Italian Version = 0.72.

The *Eating Disorder Inventory-2 (EDI-2)*, a self-administered 91-item questionnaire that evaluates symptoms and characteristics typical of patients affected with ED [51]. It consists of 11 scales: Drive to Thinness, Bulimia, Body Dissatisfaction, Inadequacy, Interpersonal Distrust, Perfectionism, Asceticism, Interoceptive Awareness, Impulsiveness, Social Insecurity, and Maturity Fears. All the scales were applied in the present research. Cronbach’s alpha for EDI-2 Italian Version = 0.85.

The *Youth Self-Report* (YSR) for ages ranging from 11 to 18 and Child Behavior Checklist (CBCL) for parents [55] measures perceived competencies, adaptive functioning and problems of adolescents within the past 6 months. They have been shown to have adequate reliability and validity [55]. The questionnaires include 132 items, of which 20 are competence items (social, activity and academic competence score) and 112 measure eight symptom subscales: withdrawn, somatic complaints, anxiety/depression (grouped into the internalizing problems cluster), aggressive behavior and rule-breaking behavior (grouped into the externalizing problems cluster) and three subscales measuring problems that are both internalizing and externalizing (thought, attention and social problems). Each item is scored on three levels (0 = not true, 1 = sometimes true, 3 = always true). The total problem scale consists of the accumulation of the scores on the 8 symptom subscales and one subscale called “other problems”. The item scores can also be converted into 6 DSM-oriented scales: anxiety, affective, somatic, conduct, oppositional-defiant and attention deficit/ hyperactivity problems. Mean scores on the YSR/CBCL subscales can be compared with the scores of normal controls of the same age and gender, obtaining a T score, that are considered in normal range (T score < 65), borderline clinical range (T score between 65 and 70) and clinical range (T score > 70). Cronbach’s alpha for YSR Italian Version = 0.81; Cronbach’s alpha for CBCL Italian Version = 0.82.

### Statistical analysis

Three diagnostic groups according to DSM-5 definition were considered: ANR, ANBP and OSFED. The prevalence of each diagnosis at each time-point was compared with the χ^2^ test. Descriptive statistics concerning DSM 5 severity levels was performed.

BMI, personality and psychopathology measures of the follow-up sample at T0 were compared with those of the whole T0 sample using bivariate one-way ANOVA to explore representativeness.

The GLM ANOVA for repeated measures was applied to compare clinical measures (BMI), personality traits (TCI), self-rated (YSR) and parent rated (CBCL) general functioning, and eating psychopathology (EDI-2) among the treatment endpoints (T0-T2). Student’s t-test for repeated measures was applied as post-hoc between treatment endpoints (T0-T1, T0-T2, T1-T2).

Based on the diagnostic evolution, participants at T2 were grouped into: (1) “healed” group (i.e. no DSM 5 criteria for an eating disorder); (2) “improved” group (i.e. diagnostic improvement with persistence of any DSM 5 diagnostic criteria); (3) “stable” group (i.e. neither improved nor worsened their diagnosis); (4) “worsened” group (i.e. worsened their diagnosis or BMI); (5) “dropped out” from treatment (i.e. between T0 and T1 or between T1 and T2). No participant worsened, hence the 4th group was empty. Only four participants improved displaying minimal residual symptoms at T2, thus they were grouped with the healed in the “improved” group. Personality and psychopathology scores at T0 were compared using the ANOVA among the improved, stable, and dropped out groups to evidence dropout predictors and medium-term prognostic factors.

Due to the non-normal distribution in the follow-up sample, available variables (BMI, TCI, EDI-2) were compared between T2 and T3 using the Wilcoxon rank test.

At T3 two subgroups were defined based on diagnostic criteria: “healed” (i.e. without any diagnostic criteria) and “non-healed” (i.e. with full or partial ED diagnosis). These were compared using the Mann-Whitney U test due to the non-normal distribution, to evidence long-term prognostic factors.

The T0-T2 differences on BMI, personality and psychopathology measures which significantly changed after treatment (deltas) were calculated and correlated with each other and with the measures at T0 using a multivariate regression analysis. A hierarchy descending from personality changes to BMI changes, through psychopathology changes, was applied. BMI deltas were used as dependent variables with respect to personality, psychopathology, and BMI at T0. Psychopathology deltas were used as dependent variables with respect to personality deltas and T0 measures. Personality deltas were used as dependent variables with respect to BMI at T0.

Statistical analysis was carried out with SPSS 27 for Windows. A Bonferroni correction was applied to the ANOVA analysis on repeated measures to reduce the type 1 error due to the high number of considered variables for each test (7 for TCI with *p <* 0.007, 11 for EDI-2 with *p <* 0.005, 17 for YSR and 16 for CBCL with *p <* 0.003). In consideration of the explorative nature of the analysis and low number of the sub-samples it was considered a *p* < 0.05 significance threshold in the analysis on the follow-up group. In consideration of the data reduction due to variable selection a *p* < 0.01 was considered acceptable for correlation and regression analysis.

## Results

### T0-T2 clinical course and diagnostic migration

At T0 47 participants (64.4%) had ANR (mean age 15.79 ± 1.32), 7 (9.6%) ANBP (mean age 14.57 ± 1.62), 19 (26%) had OSFED AN (mean age 16.11 ± 1.05).

The 4.2% had a BMI < 15 kg/m^2^, the 12.5% a BMI between 15 and 15.99 kg/m^2^, the 26.4% a BMI between 16 and 16.99 kg/m^2^ and, finally, the 56.9% a BMI > 17 kg/m^2^. The minimum BMI at T0 is 14.30 kg/m^2^, while the maximum BMI at T0 was 20.96 kg/m^2^.

Table 1 displays the diagnostic distribution of the sample at T0 and the diagnostic distribution at the following time-points.

**Table 1:** Evolution of the diagnoses across the follow-up timepoints

*Timepoints*	*T0*	*T1*	*T2*	*T3*
*Diagnoses*	*N (%)*	*N (%)*	*N (%)*	*N (%)*
*Restricter AN*	*47 (64.4)*	*15 (23.4)*	*13 (22)*	*3 (15.8)*
*Binge-Purging AN*	*7 (9.6)*	*3 (4.7)*	*1 (1.7)*	*0 (0)*
*EDws*	*19 (26)*	*46 (71.9)*	*7 (9.6)*	*2 (10.5)*
*Healthy*	*0 (0)*	*0 (0)*	*38 (64.4)*	*14 (73.7)*
*TOTAL*	*73 (100)*	*64 (87.7)*	*59 (80.8)*	*19 (26)*
***Chi-square***	*-*	*42.298*	*17.078*	*5.429*
***df***	*-*	*4*	*6*	
***P***	*-*	*0.000*	*0.009*	*0.246*

*EDws = Eating Disorders without specification; T0 = Enrollement; T1 = 6 months; T2 = 12 months; T3 = 10 years*

At T2 59 participants (81%) (mean age 17.00 ± 1.27) completed the treatment program, with an overall dropout rate of 19%. The rate of ANR diagnosis significantly reduced from T0 to T1 and from T2 to T3. The diagnosis of ANBP progressively reduced from T0 to T3.

The diagnosis of OSFED provisionally increased between T0 to T1, then significantly decreased at T2 and persisted stable at T3, due to diagnostic migration of the full AN diagnosis (Table 1).

Among the 59 participants that completed the therapeutic program, 42 (71.2%) improved their diagnosis (from full AN diagnosis to OSFED or healed), 11 (18.6%) were stable, 5 (8.5%) relapsed after an initial improvement at T1, one (1.7%) worsened her condition (from OSFED to ANR diagnosis).

Among the 42 (57.5%) participants who reached full remission, 27 (64.3%) were affected with RAN, 2 (4.8%) with BPAN, and 13 (30.9%) with OSFED at T0. Among the 17 (21.9%) who remained stable, 10 were affected with RAN (58.8%), 3 (17.6%) with BPAN and 4 (23.5%) with OSFED at T0 (chi-square = 3.857; df = 4; p < 0.426).

### Clinical course and diagnostic migration at 15-years follow-up

No significant difference was found with ANOVA between the follow-up and the whole sample recruited at T0 among personality, general functioning, and general and eating psychopathology.

Among the 19 participants to the follow-up 14 (73.7%) were healthy (mean age 28.93 ± 2.09), 2 (10.5%) had OSFED diagnosis (mean age 30.50 ± 2.12), 3 (15.8%) full diagnosis of RAN (mean age 31.00 ± 1.00). Among these 11 (57.9%) maintained their healthy condition from T2, 3 (15.8%) healed, 5 (26.3%) relapsed.

Among the 14 (73.7%) healed at follow-up, 9 (47.4%) had RAN, 1 (5.3%) had BPAN, and 4 (21%) had OSFED. Among the 5 not healed 4 (80%) had RAN, 1 (20%) had BPAN (chi-square = 2.140; df = 2; p < 0.343).

### Changes between T0 and T2 among BMI, personality and psychopathology measures in the treatment group

ANOVA for repeated measures evidenced a significant increase in BMI during treatment period (T0-T1-T2) in the whole treatment group (F = 56.813; df = 1; p < 0.001) (Table 2).

**Table 2:** BMI evolution across follow-up timepoints

*BMI scores*		*First timepoint*	*Second timepoint*	*t-test for paired samples*	
	*N*	*M ± SD*	*M ± SD*	*T*	*P*
*BMI_T0 vs. BMI_T1*	*64*	*17.47 ± 1.53*	*18.65 ± 1.54*	*-5.684*	***0.000***
*BMI_T0 vs. BMI_T2*	*59*	*17.53 ± 1.45*	*19.23 ± 1.40*	*-8.313*	***0.000***
*BMI_T1 vs. BMI_T2*	*59*	*18.73 ± 1.54*	*19.23 ± 1.40*	*-3.417*	***0.001***
		***First timepoint***	***Second timepoint***	***Wilcoxon rank test (WRT)***	
	***N***	***M ± SD***	***M ± SD***	***Test***	***P***
*BMI_T2 vs. BMI_T3*	*19*	*17.40 ± 1.41*	*20.87 ± 3.16*	*142.000*	*0.059*

*BMI = Body Mass Index; T0 = Enrollement; T1 = 6 months; T2 = 12 months; T3 = 10 years; Bold fonts indicate p ≤ 0.001*

Table 2 displays the BMI changes at each time-point and the results of post-hoc comparison with t-test for paired measures. BMI increases significantly for the treatment group at any time point (p < 0.001).

**Table 3:** Personality evolution from T0 to T2

*TCI*	*T0*	*T2*	*GLM ANOVA* *for repeated measures*	
	*N = 73*	* N = 59*		
	*M ± SD*	*M ± SD*	*F*	*P*
*Novelty Seeking*	*17.97 ± 5.27*	*19.72 ± 4.16*	*5.970*	*0.018*
*Harm Avoidance*	*18.33 ± 6.11*	*17.04 ± 5.96*	*3.751*	*0.058*
*Reward Dependence*	*15.11 ± 3.77*	*16.45 ± 2.89*	*2.088*	*0.154*
*Persistence*	*5.72 ± 1.61*	*4.79 ± 1.64*	*15.587*	***0.000***
*Self-directedness*	*25.59 ± 7.59*	*28.84 ± 6.41*	*13.164*	***0.001***
*Cooperativeness*	*32.38 ± 5.98*	*33.96 ± 4.47*	*1.045*	*0.311*
*Self-transcendence*	*13.72 ± 4.67*	*12.76 ± 3.95*	*5.115*	*0.027*

*TCI = Temperament and Charatcer Inventory; T0 = Enrollement; T1 = 6 months; T2 = 12 months; T3 = 10 years; Bold fonts indicate p ≤ 0.007*

Table 3 displays personality changes from T0 to T2. ANOVA for repeated measures evidenced a significant reduction in persistence (*p* < 0.001), and increase in self-directedness (*p* < 0.001).

**Table 4:** Eating psychopathology evolution across follow-up timepoint

*EDI-2*	*T0*	*T1*	*T2*	*GLM ANOVA*		*t-test for paired samples*					
	*N = 73*	* N = 64*	* N = 59*	*T0-T1-T2*		*T0-T1*		*T1-T2*		*T0-T2*	
	*M ± SD*	*M ± SD*	*M ± SD*	*F*	*P*	*T*	*P*	*t*	*P*	*t*	*P*
*Drive to Thinness*	*9.97 ± 6.85*	*4.92 ± 4.93*	*4.77 ± 6.06*	*8.811*	***0.004***	*2.751*	*0.008*	*0.324*	*0.747*	*2.968*	***0.004***
*Bulimia*	*1.34 ± 2.08*	*1.48 ± 2.59*	*1.38 ± 2.82*	*0.147*	*0.703*	*-0.971*	*0.335*	*0.537*	*0.593*	*-0.383*	*0.703*
*Body Dissatisfaction*	*6.37 ± 5.88*	*6.57 ± 5.79*	*6.30 ± 7.44*	*0.066*	*0.798*	*0.007*	*0.995*	*0.836*	*0.406*	*0.257*	*0.798*
*Inadequacy*	*5.60 ± 5.65*	*5.29 ± 4.25*	*4.26 ± 4.83*	*3.613*	*0.062*	*0.173*	*0.863*	*2.421*	*0.019*	*1.901*	*0.062*
*Perfectionism*	*3.69 ± 3.22*	*3.43 ± 2.33*	*2.43 ± 2.49*	*4.859*	*0.031*	*0.253*	*0.801*	*2.718*	*0.009*	*2.204*	*0.031*
*Interpersonal Distrust*	*4.73 ± 4.41*	*4.45 ± 3.68*	*3.69 ± 4.19*	*1.276*	*0.263*	*-0.330*	*0.743*	*1.253*	*0.215*	*1.130*	*0.263*
*Interoceptive Awareness*	*5.56 ± 5.76*	*4.57 ± 4.61*	*2.84 ± 3.77*	*11.87*	***0.001***	*0.787*	*0.434*	*3.357*	***0.001***	*3.446*	***0.001***
*Maturity Fears*	*6.11 ± 4.89*	*5.76 ± 3.37*	*5.39 ± 4.13*	*3.267*	*0.076*	*0.747*	*0.458*	*0.658*	*0.513*	*1.807*	*0.076*
*Asceticism*	*4.84 ± 3.75*	*4.54 ± 2.93*	*3.80 ± 3.01*	*1.906*	*0.173*	*-0.122*	*0.904*	*1.965*	*0.054*	*1.381*	*0.173*
*Impulsivity*	*3.34 ± 4.37*	*2.75 ± 2.96*	*1.64 ± 2.48*	*7.971*	*0.007*	*0.549*	*0.585*	*2.936*	***0.005***	*2.823*	*0.007*
*Social Insecurity*	*5.23 ± 4.03*	*4.12 ± 3.06*	*3.53 ± 3.18*	*6.893*	*0.011*	*1.515*	*0.135*	*1.861*	*0.068*	*2.626*	*0.011*

*EDI-2 = Eating Disorder Inventory-2; T0 = Enrollement; T1 = 6 months; T2 = 12 months; T3 = 10 years; Bold fonts indicate p ≤ 0.005*

Table 4 displays eating psychopathology changes from T0 to T2 in the treatment group. Two psychopathology traits significantly improved during the whole treatment: drive for thinness (*p* < 0.004) and interoceptive awareness (*p* < 0.001).

**Table 5:** General CBCL psychopathology evolution across follow-up timepoints

*CBCL*	*T0*	*T1*	*T2*	*GLM ANOVA*		*t-test for paired samples*					
	*N = 73*	* N = 64*	* N = 59*	*T0-T1-T2*		*T0-T1*		*T1-T2*		*T0-T2*	
	*M ± SD*	*M ± SD*	*M ± SD*	*F*	*P*	*T*	*P*	*t*	*P*	*t*	*P*
*Activities*	*4.37 ± 1.78*	*4.06 ± 1.64*	*3.77 ± 1.59*	*5.895*	*0.018*	*1.207*	*0.232*	*1.683*	*0.098*	*2.428*	*0.018*
*Social*	*5.07 ± 1.53*	*4.80 ± 1.05*	*4.75 ± 1.09*	*2.898*	*0.094*	*1.140*	*0.258*	*0.281*	*0.780*	*1.702*	*0.094*
*School*	*5.45 ± 0.67*	*5.24 ± 0.53*	*5.19 ± 0.65*	*10.110*	***0.002***	*3.083*	*0.003*	*0.404*	*0.688*	*3.180*	***0.002***
*Withdrawn*	*5.39 ± 3.30*	*4.73 ± 2.39*	*4.33 ± 3.18*	*4.789*	*0.033*	*1.470*	*0.147*	*0.799*	*0.427*	*2.188*	*0.033*
*Somatic Complaints*	*2.58 ± 2.27*	*2.70 ± 1.55*	*1.98 ± 1.53*	*4.101*	*0.047*	*-0.680*	*0.499*	*2.767*	*0.008*	*2.025*	*0.047*
*Anxious/depressed*	*8.78 ± 4.74*	*7.46 ± 2.72*	*6.67 ± 4.01*	*14.479*	***0.000***	*1.947*	*0.056*	*1.834*	*0.072*	*3.805*	***0.000***
*Internalizing Problems*	*16.22 ± 7.93*	*14.00 ± 5.48*	*12.44 ± 6.77*	*15.944*	***0.000***	*2.407*	*0.019*	*1.399*	*0.167*	*3.993*	***0.000***
*Social Problems*	*1.47 ± 1.64*	*1.86 ± 1.45*	*1.58 ± 1.54*	*0.085*	*0.772*	*-1.849*	*0.069*	*1.696*	*0.095*	*-0.291*	*0.772*
*Thought Problems*	*1.29 ± 1.64*	*1.03 ± 1.14*	*0.77 ± 0.93*	*7.141*	*0.010*	*1.122*	*0.266*	*1.692*	*0.096*	*2.672*	*0.010*
*Attention Problems*	*3.68 ± 2.89*	*3.19 ± 1.91*	*3.33 ± 2.43*	*3.084*	*0.084*	*2.084*	*0.041*	*-0.595*	*0.554*	*1.756*	*0.084*
*Delinquent Behavior*	*1.28 ± 1.28*	*1.25 ± 1.04*	*1.21 ± 1.41*	*0.307*	*0.582*	*0.495*	*0.623*	*0.265*	*0.792*	*0.554*	*0.582*
*Aggressive Behavior*	*5.58 ± 3.85*	*5.23 ± 3.12*	*4.69 ± 3.34*	*3.994*	*0.050*	*0.943*	*0.349*	*1.527*	*0.132*	*1.998*	*0.050*
*Externalizing Problems*	*6.91 ± 4.63*	*6.86 ± 3.74*	*5.90 ± 4.32*	*4.281*	*0.043*	*0.275*	*0.784*	*2.150*	*0.036*	*2.069*	*0.043*
*Total Problems*	*34.35 ± 15.97*	*30.25 ± 12.63*	*27.81 ± 15.44*	*12.995*	***0.001***	*2.384*	*0.020*	*1.194*	*0.237*	*3.605*	***0.001***
*Total competence*	*14.75 ± 2.49*	*14.10 ± 2.30*	*13.83 ± 2.37*	*9.356*	***0.003***	*1.825*	*0.073*	*0.976*	*0.333*	*3.059*	***0.003***

*CBCL = Child Behavior Cecklist; T0 = Enrollement; T1 = 6 months; T2 = 12 months; T3 = 10 years; Bold fonts indicate p ≤ 0.003*

Table 5 displays general psychopathology changes from T0 to T2 in the treatment group scored by parents at CBCL. Anxious/Depressed (*p* < 0.001), Internalizing (*p* < 0.001), and Total problems (*p* < 0.001) decreased, as far as School functioning (*p* < 0.002) and Total competence (*p* < 0.003).

**Table 6:** General YSR psychopathology evolution across follow-up timepoints

*YSR*	*T0*	*T1*	*T2*	*GLM ANOVA*		*t- test for paired samples*					
	*N = 73*	* N = 64*	* N = 59*	*T0-T1-T2*		*T0-T1*		*T1-T2*		*T0-T2*	
	*M ± Ds*	*M ± Ds*	*M ± Ds*	*F*	*P*	*t*	*P*	*t*	*P*	*t*	*P*
*Activities*	*3.94 ± 1.32*	*3.41 ± 1.00*	*3.60 ± 1.31*	*2.068*	*0.156*	*2.267*	*0.027*	*-0.804*	*0.425*	*1.438*	*0.156*
*Social*	*5.39 ± 1.64*	*5.22 ± 1.25*	*5.74 ± 2.91*	*0.893*	*0.349*	*0.279*	*0.781*	*-1.452*	*0.152*	*-0.945*	*0.349*
*Academic Performance*	*2.35 ± 0.45*	*2.38 ± 0.34*	*2.27 ± 0.33*	*1.121*	*0.294*	*-0.707*	*0.482*	*1.646*	*0.105*	*1.059*	*0.294*
*Withdrawn*	*5.62 ± 2.97*	*4.53 ± 2.12*	*4.22 ± 2.56*	*13.871*	***0.000***	*2.524*	*0.014*	*0.598*	*0.552*	*3.724*	***0.000***
*Somatic Complaints*	*3.62 ± 2.40*	*3.38 ± 1.77*	*2.70 ± 1.85*	*7.211*	*0.009*	*0.769*	*0.445*	*1.920*	*0.060*	*2.685*	*0.009*
*Anxius/depressed*	*11.32 ± 5.32*	*9.33 ± 4.06*	*8.33 ± 4.65*	*18.088*	***0.000***	*2.734*	*0.008*	*1.719*	*0.091*	*4.253*	***0.000***
*Internalizing Problems*	*19.62 ± 8.32*	*16.81 ± 6.09*	*14.68 ± 7.60*	*22.029*	***0.000***	*2.402*	*0.019*	*2.199*	*0.032*	*4.693*	***0.000***
*Social Problems*	*2.95 ± 2.36*	*2.97 ± 2.10*	*2.30 ± 2.27*	*5.070*	*0.028*	*-0.511*	*0.611*	*2.626*	*0.011*	*2.252*	*0.028*
*Thought Problems*	*2.33 ± 2.39*	*1.58 ± 1.68*	*1.47 ± 1.54*	*8.881*	*0.004*	*2.471*	*0.016*	*0.258*	*0.798*	*2.825*	*0.006*
*Attention Problems*	*5.64 ± 2.99*	*5.33 ± 2.31*	*4.78 ± 2.87*	*7.983*	*0.006*	*1.220*	*0.227*	*2.107*	*0.039*	*0.450*	*0.006*
*Delinquent Behavior*	*1.90 ± 2.16*	*1.81 ± 1.07*	*1.70 ± 1.67*	*0.203*	*0.654*	*-0.063*	*0.950*	*0.503*	*0.617*	*0.450*	*0.654*
*Aggressive Behavior*	*7.91 ± 4.07*	*8.28 ± 3.02*	*6.58 ± 3.49*	*10.831*	*0.002*	*-0.756*	*0.452*	*4.312*	***0.000***	*3.291*	*0.002*
*Externalizing Problems*	*9.51 ± 5.44*	*9.72 ± 3.95*	*8.39 ± 4.63*	*3.855*	*0.054*	*-0.391*	*0.697*	*2.359*	*0.022*	*1.963*	*0.054*
*Total Problems*	*45.78 ± 19.10*	*41.47 ± 12.81*	*36.48 ± 16.72*	*16.962*	***0.000***	*1.654*	*0.103*	*2.826*	*0.006*	*4.118*	***0.000***
*Total competence*	*11.71 ± 2.39*	*11.06 ± 1.80*	*11.32 ± 2.18*	*1.192*	*0.279*	*1.595*	*0.116*	*-1.034*	*0.305*	*1.092*	*0.279*

*YSR = Youth Self-report; T0 = Enrollement; T1 = 6 months; T2 = 12 months; T3 = 10 years; Bold fonts indicate p ≤ 0.001*

Table 6 displays general psychopathology changes from T0 to T2 in the treatment group scored by participants at YSR. Withdrawn (*p* < 0.001), Anxious/Depressed (*p* < 0.001), Internalizing (*p* < 0.001), and Total problems (*p* < 0.001) significantly decreased.

### Changes between T2 and T3 among BMI, personality and psychopathology measures in the follow-up group

Wilcoxon ranking test of variance evidenced no significant increase in BMI after treatment period (T2 to T3) in the follow-up group (*p* < 0.059) (Table 2).

Among personality dimensions Persistence (M = 4.79, SD = 1.64 vs. M = 5.79 SD = 1.81; WRT = 126.000; *p* < 0.019), Self-directedness (M = 28.84 SD = 6.41 vs. M = 36.16, SD = 5.65; WRT = 162.000; *p* < 0.007), and Cooperativeness (M = 33.96, SD = 4.47 vs. M = 36.63 SD = 2.54; WRT = 97.500; p < 0.033) displayed a significant increase between T2 and T3.

Among EDI-2 psychopathology measures no item displayed significant changes between the two time-points.

### T0 comparison between the outcome subgroups

**Table 7:** ANOVA comparison of T0 features between clinical evolution subgroups at T2

*Measure*	*Dropout (a)*	*Stable (b)*	*Improved (c)*	*F*	*P*	*Post-hoc*
*Reward Dependence*	*12.51 ± 4.59*	*15.06 ± 3.19*	*16.00 ± 3.33*	*5.031*	***0.009***	*c > a*
*Cooperativeness*	*28.39 ± 6.59*	*31.88 ± 5.55*	*32.38 ± 5.98*	*5.073*	***0.009***	*c > a*
*Aggressive-YSR*	*7.50 ± 4.36*	*10.00 ± 4.11*	*7.05 ± 3.77*	*3.409*	***0.039***	*c > b*
*Externalizing-YSR*	*9.71 ± 6.12*	*12.18 ± 5.92*	*8.37 ± 4.70*	*3.155*	***0.049***	*c > b*
*Delinquent-CBCL*	*1.14 ± 1.23*	*2.00 ± 1.27*	*1.03± 1.22*	*3.829*	***0.026***	*c > b*

*CBCL = Child Behavior Checklist; YSR = Youth Self-report; Bold fonts indicate p ≤ 0.05*

Table 7 displays the ANOVA comparison of T0 clinical features between clinical evolution subgroups at T2 (Dropout, Stable and Improved). The Improved group displays higher reward dependence (*p* < 0.009) and cooperativeness (*p* < 0.009) with respect to the dropout. The Improved group displays lower self-reported (YSR), aggressiveness (*p* < 0.039) and externalizing (*p* < 0.049) symptoms and lower parent-reported (CBCL) delinquent symptoms (*p* < 0.026) with respect to the stable group.

### Mann-Withney U test comparison between FU subgroups

The Mann-Whitney U test comparison between healed and not healed participants at T3 evidenced a higher maturity fear among the non-healed participants (U = 57.000; p < 0.044).

### Multivariate correlation analysis between BMI, personality and psychopathology changes

Multivariate correlation analysis evidenced that both the changes in BMI between T0 and T1 (p < 0.001) and those between T0 and T2 (p < 0.001) were negatively correlated to BMI at T0. The changes in BMI between T0 and T2 were negatively correlated to the T0 values in inadequacy (p < 0.008) and interpersonal distrust (p < 0.002). Those between T0 and T1 also displayed a trend towards significance for a negative correlation with interpersonal distrust (p < 0.018) and those between T1 and T2 with maturity fears (p < 0.018).

Multivariate analysis of BMI changes with personality and psychopathology changes evidenced a positive between T0-T1 BMI increase and the increase in self-directedness (p < 0.014), and the T0-T1 reduction in impulsiveness (p < 0.007).

Multivariate analysis among personality and psychopathology changes evidenced a correlation between the T0-T2 changes in self-directedness and those in interoceptive awareness (p < 0.043).

Among eating psychopathology, the T0-T2 changes of interoceptive awareness were significantly related to YSR changes in withdrawal (p < 0.000), anxious/depressed symptoms (p < 0.003) and internalizing problems (p < 0.004). T0-T2 changes in impulsiveness were related to YSR changes in withdrawal (p < 0.003).

### Psychopathology predictors of change after treatment at T0

Multivariate analysis between personality and psychopathology changes and T0 measures evidenced that T0 body dissatisfaction was positively related to the T0-T2 changes in CBCL school performances (p < 0.013) and total competencies (p < 0.009). T0 perfectionism was positively related to YSR academic performance (p < 0.001). T0 interoceptive awareness was positively related with its T0-T2 changes (p < 0.001). T0 asceticism was positively related with YSR changes in withdrawal (p < 0.008). T0 impulsivity was positively related to the T0-T2 changes in YSR total competencies (p < 0.006).

T0 novelty seeking (p < 0.013) and harm avoidance (p < 0.010) were positively related to T0-T2 changes in CBCL total competencies. T0 Cooperativeness (p < 0.001) and self-transcendence (p < 0.001) were positively correlated to the T0-T2 changes in CBCL total competencies.

T0 thought problems at YSR (p < 0.002), and CBCL activities (p < 0.006), social problems (p < 0.001), withdrawal (p < 0.001), somatization (p < 0.001), anxiety (p < 0.001), internalizing problems (p < 0.001), thought problems (p < 0.008) and total competencies (p < 0.002) were positively related to T0-T2 changes in CBCL total competencies.

## Discussion

The present study assessed 73 adolescent participants affected with AN and OSFED AN who underwent a multidisciplinary treatment program, and followed-up some of them 15 years after the completion of the treatment.

### Treatment drop-out

The dropout rate was 19%, but only 5 subjects (7%) received less than 6 months of treatment. Our dropout rate is better than that reported in the literature [11–13] ranging from 22 to 42%, but it could be partly biased by the retrospective file selection for the recruitment. Lower reward dependence and cooperativeness characterized the AN participants who dropped out. Both these personality traits are strictly related to the skills needed to build a stable and trusting therapeutic relationship [5, 56]. In fact, cooperativeness is a dropout predictor for psychotherapy in adults with AN [57, 58]. Reward dependence is a dropout predictor in the treatment of drug-dependence [59]. Both are predictors of treatment response in depressed patients [60, 61]. This is the first evidence of their correlation with the dropout of adolescent with AN. Moreover, according to Agüera and coworkers [62], low reward dependence is a more specific predictor for adolescents with respect to cooperativeness.

### Clinical course and diagnostic migration after treatment

A high rate (64%) of study treatment sample reached complete remission, consistent to the best literature outcomes reported in outpatient [32] settings. This confirms the efficacy of a multidisciplinary approach including the B-APP [50] as a brief psychodynamic psychotherapy, which is thus represent the highest-standard available [63]. As a difference from Rosling and coworkers [32] who had evidenced a good outcome for 20% of the AN and 48% of the OSFED patients, our study did not evidence a significantly better outcome for the OSFED with respect to the full-syndrome AN. This implies that B-APP displays a good efficacy for both, as for adults with AN [13]. This is probably consequent to the tailored approach of this psychodynamic psychotherapy with respect to the more standardized approaches of CBT or other psychotherapies applied to AN.

### Changes in BMI, personality traits and psychopathology after multimodal treatment

BMI significantly increased after each time-point, reaching normal weight at the end of the treatment. Treatment also produced a significant reduction of the trait of persistence and an increase in that of self-directedness. Persistence is a temperament trait favoring fasting perseverance in AN subjects [5]. Past research evidenced that it is related to unexpressed creative skills [64]: in fact psychotherapy activated creative copying, which favored its reduction. Self-directedness belongs to the psychopathological core of AN [65, 66], and is related to frailty in the Self, which is a typical feature of AN [67–69]. It typically increases with physiological character development [56] and with psychotherapy treatments [13, 70], and implies an improvement of the Self organization.

Interoceptive awareness significantly decreased between T0 and T1, and between T0 and T2. It measures the degree of confusion that the ED patients perceive with respect to their bodily sensations, their emotions, and their feelings [52]. Along with the psychosomatic interpretation of AN [71] the improvement of interoceptive awareness reached with the treatment displays the progressive integration of cognitive, emotional, relational, and somatosensory functions of the Self [68, 69, 72] which discards the eating symptom.

Drive to thinness is a core psychopathology trait in AN, and decreases after treatment also in adults with AN [13]. Decrease in drive to thinness corresponds to the reduction of the need of being thin to control deep anguish [71]. Its improvement is coupled with the reduction of impulsivity in the second part of the treatment, and is possibly consequent to the strengthening of the Self obtained with psychotherapy [68, 69, 72] .

Both parents and AN participants report a substantial improvement in both anxious and depressive symptoms, and internalizing and total problems after treatment. According to its theoretical presuppositions [50], B-APP does not only address AN itself, but also provides an overall improvement in adolescent’s wellbeing [50]. As a discrepancy, while parents reported a decrease in their daughters’ school performance and general competencies, the AN adolescents reported a reduction of withdrawal without sensible reduction in school performance and competencies. This leads to a double interpretation: the improvement of social competencies may be coupled with a reduction of the investment in performance, with a rebalancing towards the relational side of the self, consequent to treatment [73]. Nevertheless, the discrepancy may also underline the divergence between the performance-focused attitudes of parents with respect to the relational attitudes of their daughters. This may suggest parental neglect towards the relational needs of the daughters, which may influence the pathogenesis or maintenance of the AN [74].

### Predictors of resistance and change

In AN adults, lower harm avoidance [75], body dissatisfaction, and binge-eating attitudes predicted healing [13]. As a difference, in the healed group of our sample, self-rated aggressive and externalizing symptoms, and parent-rated delinquent behaviors were significantly lower at T0 than in the stable one. This suggests that in adulthood the outcome predictors are more related to psychopathology, while in adolescence they are more relevant predictors of those traits which interfere with the relationships, including also the therapeutic one [46]. According to theoretical models based on attachment [5, 69] this finding suggests that favoring a trusting relationship with caregivers and therapists may represent a priority for addressing resistances in AN adolescents [67, 76].

### Predictors of outcome and mechanisms of change

As previously evidenced [7], the initial lower BMI is a negative predictor of therapeutic efficacy. The degree of BMI loss seems related to the degree of underdevelopment of the Self [68, 74]. In fact the changes in BMI in the first phase of treatment correlate with the overall rise in self-directedness, a behavioral measure of the integration of the Self [56].

BMI increase in the initial phase of treatment also relates to lower interpersonal distrust and inadequacy at T0. This suggests that building a solid therapeutic alliance since the beginning of the treatment favors rapid clinical improvement [5, 77].

The T0-T2 improvement of interoceptive awareness relates to the improvement in anxious/depressive symptoms, internalizing problems, and social withdrawal. A better interoceptive awareness at intake also predicts greater improvement of emotional awareness after treatment. This supports the prognostic relevance of interoceptive awareness for treatment outcome, and supports that this trait is a relevant target for an effective brief psychodynamic psychotherapy in AN adolescents [50, 71].

The BMI increase between T0 and T1 correlates with the reduction of impulsiveness. This suggests that the better integration of emotions in the Self, related to the improvement in interoceptive awareness, favors both BMI changes and emotional control [15]. Impulsiveness improvement also favors the reduction of social withdrawal suggesting that emotion regulation favors the copying with relationships creating a virtuous circle [15, 46].

Body dissatisfaction is a core feature of AN. It directly relates to attachment dynamics [78], and predicts AN treatment outcome [13]. Its correlation with worsening in school performance is unexpected, and supports the hypothesis that the improvement in social competencies is balanced by a lesser concentration on school performance [73]. Pathological perfectionism is a well-known feature of AN [79], conceptually related with the personality trait of persistence [56]. The fact that higher initial perfectionism predicts changes in persistence is not surprising, and may be consequent to the better copying of the AN adolescent with her emotional needs during treatment [5, 50]. Finally, the relationship between initial asceticism and improvement in social withdrawal links the tendency to self-punitive attitudes with social retirement. No previous evidence supports this hypothesis, which needs confirmation by further research.

The extensive correlations of changes in parent-rated total competencies with T0 adolescent-rated personality and psychopathology suggests a bias, in fact adolescent’s characteristics may influence parental evaluation of competencies after treatment, rather than being predictors of change.

### 15-years follow-up changes

Although small the follow-up group had a good representativeness with respect to the initial sample.

As already evidenced, the rate of complete remission increased at the 15-year follow-up [12, 16] reaching 74%, which is the best reported in the literature [6, 80–82]. This suggests that, as observed in adults, also adolescents with AN sometimes need a long time to heal [23, 24]. As a difference from previous reports, the initial diagnosis did not represent a strong prognostic factor of long-term improvement [32, 80].

An overall trend toward an increase in BMI was observed at the long-term follow-up that however did not reach statistical significance due the small sample size, but in the majority of participants BMI changes were stable or improving over time [80, 81].

Levels of persistence increased suggesting a return to the levels which are partly genetically determined since birth [56], without influencing the final outcome.

Self-directedness and cooperativeness both increased at T3. Even though low cooperativeness is a dropout predictor [57, 58], and a favorable prognostic factor for psychotherapy [60, 61], it did not increase significantly at T2, and it was neither a target of psychotherapy, nor a necessary change for treatment outcome. It is thus possible that the multimodal treatment unblocked the physiologic character development as a whole, facilitating the post-treatment increasing of both self-directedness and cooperativeness, along with a modest increasing of self-transcendence, supporting the evidence that psychodynamic psychotherapy works after the end of the treatment [63, 83].

## Conclusion

The present outcome study supports the treatment efficacy and long-term results stabilization of outpatient multidisciplinary treatment for adolescents with AN including a brief psychodynamic psychotherapy [50, 63]. It confirms the initial BMI, baseline inadequacy, and interpersonal distrust as predictors of BMI improvement, and lower reward dependence and cooperativeness as predictors of dropout. Reduction in persistence and rise in self-directedness accompany treatment evolution. The increase in self-directedness and the decrease in impulsiveness were correlated with the increase in BMI increase. This suggests that the dynamics linking psychotherapeutic treatment to clinical outcome are complex and mediated by emotional integration and changes in interoceptive awareness. In fact, initial interoceptive awareness predicts emotional integration after treatment, and relates to changes in general psychopathology. Initial body dissatisfaction, perfectionism and asceticism also relate to treatment outcome. All character dimensions that developed after treatment interruption could be due to a long-lasting effect of psychodynamic psychotherapy on character development [63].

With respect to adult with AN, the treatment of adolescents with AN and its long-term outcome are more influenced by those personality and psychopathology traits which hamper the relationship with caregivers and therapists. These features represent relevant targets for treatment selection or priming, and for the treatment of resistant subjects.

### Limitations

The main limitations of the present study concern the relatively small number of subjects, in particular in the long-term follow-up group. This reduces the possibility of statistical exploration concerning clinical subgroups, and may have reduced also the strength of some statistical analysis. The results obtained by the follow-up of the study should considered cautiously and need replication, also because of the possible life events that may have influenced the outcome. Due to the natural follow-up of the study, possible involuntary recruitment biases and parental overprotection affected the recruited sample. Moreover, as evidenced by the results, some healed subjects dropped out in the long term. Nevertheless, the assessment of personality and psychopathology features permitted a better insight in the processes of treatment, adding new evidence to existing literature.

## Data Availability

The dataset generated and analyzed during this study are stored on a database of University of Turin on which FA (first author) and LA have complete control. They will be available upon reasonable request.

## Declarations

### Copyright transfer

The authors transfer the copyright to Springer Nature.
